# Virus detection using bio-based analysis systems: a review of biorecognition strategies

**DOI:** 10.55730/1300-0527.3481

**Published:** 2022-08-02

**Authors:** Nur Melis KILIÇ, Dilek ODACI DEMİRKOL

**Affiliations:** Department of Biochemistry, Faculty of Science, Ege University, İzmir, Turkey

**Keywords:** Bio-based detection technologies, point-of-care, biofunctional surface, viruses, nanotechnology, nanobiotechnology, nanomaterials

## Abstract

Infectious illnesses are on the rise in today’s world, with serious consequences for animals, plants, and humans. Several infections, including the human immunodeficiency virus, affect a large number of individuals in various countries, particularly in the poorer portions of contemporary society, and continue to cause a variety of health problems. Viruses are tiny parasitic organisms. They are infectious agents that can only reproduce within a live cell of an organism. Viruses may infect any living organism. For clinical point-of-care applications, early detections for harmful agents such as bacteria, viruses are critical. The possibility of worldwide epidemics as a result of viral propagation emphasizes the importance of creating speedy, precise, and sensitive early detection systems. Furthermore, because certain viruses have a long latent phase and can evolve from one person to another, early detection during the incubation period is critical for improving recovery rates and avoiding pandemics. Nowadays, there has been various bio-based detection systems that have rapid reaction times, user-friendly, cost-effective, and repeatable. In this review, biological molecule-based detection technologies which focus on virus analysis are examined.

## 1. Introduction

### 1.1. Biosensors

**Figure 1:**
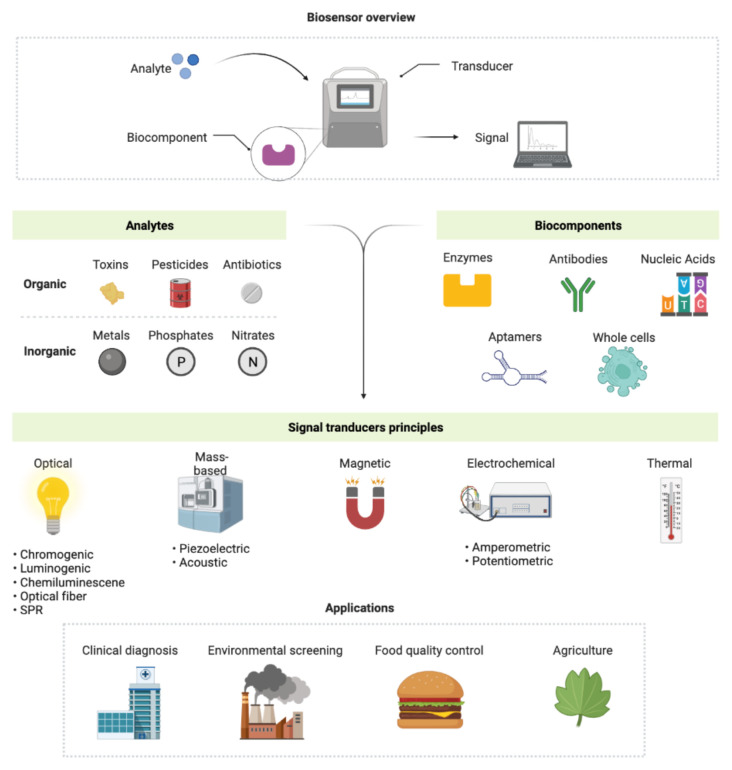
A biosensor’s overview.

For medical and biological applications, monitoring biological or biochemical processes is critical [1]. The latest increase in nanobiotechnology, according to current research trends, has given a rise to great diagnostic and therapeutic techniques such as sensors for medical and biological applications [2]. A sensor is a device or module that detects changes in physical quantities such as pressure, heat, humidity, movement, force, and electrical quantities such as current and transforms them into signals that can be observed and analyzed (Figure 1) [3]. The International Union of Pure and Applied Chemistry (IUPAC) defines a biosensor as an integrated biomolecule-transducer apparatus capable of providing specific analytical data applying a biological sensing element [4]. Biosensors are extremely selective because of their ability to control the interaction of chemicals with the immobilizing process of biological sensing components on the substrate that have a specific recognition affinity to the target [5]. An ideal sensor should possess certain characteristics, such as range, drift, calibration, sensitivity, selectivity, linearity, high resolution, reproducibility, repeatability, and response time [6]. Biosensors are devices that measure biological signals and convert them into signals. The basic elements of any biosensor are its bioreceptor, transducer, analyte, and display (Figure 1). A bioreceptor, also known as a biological recognition element, is a biological entity that produces a quantifiable signal by reacting precisely with the analyte. A transducer is a device that converts energy from one form to another [7]. High sensitivity, easy instrumentation, cost efficiency, and downsizing capability are all benefits of biosensors with an electrochemical transducer, which are employed for microliter sample volume [8]. While designing a biosensor, a balance should be achieved between the stability, costs, analyzing time, target selectivity, and quality of the analytical signal (Figure 1). Biosensors have advanced in a variety of sectors and applications, including DNA modification, drug research, security, clinical diagnostics, healthcare monitoring, food quality management, and environmental monitoring [9].

Biosensors are great low-cost, portable instruments for detecting infections, proteins, and other analytes quickly [10]. Using an interdisciplinary approach mix of methodologies from chemistry, biochemistry, nanotechnology and medical research, biosensor technologies have the ability to meet these criteria [11]. Recent biosensor developments have surprisingly demonstrated an increase in rapid and easy point-of-care detection in the biomedical area, reducing the reliance on diagnosis by hospitals and medical practitioners [12,13]. Using of nanomaterials in biosensor development has opened wide window to improve their performance characteristics such as sensitivity, stability, repeatability and reproducibility, etc. Numerous of nanomaterials such as carbon nanotubes [14], colloidal nanoparticles [15,16], dendrimers [17–19] and nanofibers, etc., [20–24] have been a great attention.

#### 1.1.1 Classification of biosensors

**Figure 2:**
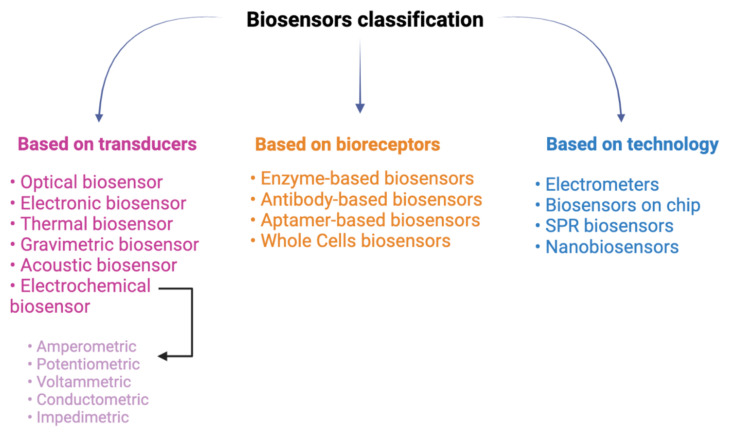
Classification of biosensors.

Bioreceptors are considered the primary component in biosensor construction [25–27]. Based on the bioreceptor, biosensors are classified as enzymatic biosensors (most common biosensor class), immunosensors (possess high specificity or sensitivity and are specifically useful in diagnosis), aptamer or nucleic acid-based biosensors (possess high specificity for microbial strains and nucleic acid-containing analyte), and microbial or whole-cell biosensors. The second classification is made on the basis of the transducer and biosensors are categorized as electrochemical (which is grouped as potentiometric, amperometric, impedimetric and conductometric), thermal, optical, and mass-based or gravimetric. Another classification includes bioreceptor-analyte combinations. Biosensors based on DNA, glucose, toxins, mycotoxins, medicines, or enzymes, etc., might be developed depending on the type of detected analyte [28, 29] (Figure 2).

### 1.2. Biosensors on virus detection

Virus infections are a significant threat to public health and the global economy. Viral diseases spread quickly through polluted water, food, and/or body fluids, killing humans and animals across the world [30, 31]. A quick, sensitive, and selective technique to early virus detection is necessary for an efficient response to viral infections. The ability to identify individual viruses with high sensitivity has a significant impact on health care since it helps clinicians to diagnose viral illnesses at an early stage. Rapid detection of a virus using biomarkers on a tiny device for illness diagnosis is crucial for public health, since it demands good clinical results. Traditional in vitro testing for viral infectious illnesses takes time and necessitates well-equipped laboratories, highly trained workers, and heavy equipment. Biosensor-based machineries can produce point-of-care devices that meet or outperform conventional standards in terms of time, precision, and cost, thanks to recent advancements in multiplexing miniaturized diagnostic technologies. Modern biosensors, which are broadly classified, combine nano- and microfabrication technologies with a variety of sensing techniques, including mechanical, optical, and electrical transducers [32].

Biosensor applications now range from the detection of tiny molecules to the assessment of pharmaceutical analytes. An ideal biosensor might give precise data for monitoring the existence of a bioprocess or deciphering its function. As a result, developing high-performance biosensors in live cells or even living bodies is critical [33]. Biosensors for infectious and pathogen illnesses have become a major subject of medical study as their usefulness in environmental studies, health care. Each year, 340 million new infections due to sexually transmitted diseases in worldwide are reported each year (2008) (Chief Medical Officer annual report 2011) [34]. Biosensors can also be used for detecting virus-mediated infections, which are divided into three categories: (i) antibodies, (ii) nucleic acids, and (iii) aptamers [35].

Nucleic acid-based tests, such as reverse transcriptase quantitative polymerase chain reaction (PCR) (RT-qPCR) and DNA microarrays, are currently the primary diagnostic procedures for the identification of inaccessible infection caused by pathogenic viruses and bacteria. These nucleic acid-based assays target DNA/RNA sequences retrieved from microorganisms in clinical samples, amplify these sequences with specific probes, and detect copies of amplified sequences quantitatively for precise bacterial/viral infection detection. These tests need the use of expensive laboratory equipment, reagents, or assay kits, as well as highly qualified specialists to conduct them. Unfortunately, such technological techniques are just unsustainable in many parts of the world. As a result, it has become a routine practice to collect and transfer biological samples to the nearest clinical laboratory facility or hospital capable of executing such treatments. Furthermore, the results may take several days to a week to reach the physician, and only then could the physician start correct therapy. Situations like those that occur during disease outbreaks or pandemics make diagnosis and treatment even more difficult. The inefficiency of current methods necessitates the creation of a reliable diagnostic procedure that can be utilized in remote medical clinics that lack the resources to perform traditional diagnostic tests. Current methods incorporate the use of nanomaterials (NMs) in diagnostics, with a focus on leveraging NMs’ unique physicochemical features for biosensing of molecular recognition with increased speed, sensitivity, and specificity. Using the microfluidics platform in conjunction with NM-mediated biosensing protocols has the potential to address future point-of-care devices that may be utilized in remote locations without relying on centralized clinical laboratories [36]. Currently, developing a quick testing kit for viral detection is an outstanding example of biosensor technology. Most used tests, such as Real Time-Polymerase Chain Reaction (RT-PCR), tend to yield false positive or negative findings owing to fluctuations in antibody production in human bodies, thus a quick testing kit for viruses is essential [9]. For this reason, the use of biosensors is necessary for virus detection.

#### 1.2.1 Biodetection technologies for SARS-CoV-2 virus

COVID-19 (coronavirus disease 2019) is a novel human viral illness that causes severe respiratory difficulty. A wave of pneumonia with an unknown etiology was recorded in China, Wuhan-Hubei Province, in December 2019 (World Health Organization (WHO) reports 2020) [37]. Coronaviruses are part of the *Coronaviridae* class, which is part of the *Nidovirales* orders, and are known to cause respiratory and intestinal illness in a variety of the avian species and mammalian. Human coronaviruses (HCoVs) are coronavirus species that have been found to infect people. This was a new type of coronavirus illness (SARS-CoV-2) which is causing severe acute respiratory sickness and a novel coronavirus disease (COVID-19), according to high-throughput sequencing [38, 39].

The development of rapid and reliable tests for COVID-19 diagnosis has a crucial role to prevent further infections to reach a pandemic control [14,17]. Although RT-PCR is currently the gold standard for detecting SARS-CoV-2, antigen rapid detection techniques are frequently employed to identify viral proteins and, while less sensitive than molecular testing, offer the advantages of being relatively affordable and providing a quick answer at the point-of-care [40,41]. The majority of them are based on immunochromatographic lateral flow assays that meet the WHO’s ASSURED (affordable, sensitive, specific, user-friendly, rapid, and robust, equipment-free, and deliverable to end users) criteria for ideal tests that can be used at all levels of the health care system, which were established in 2003 [42].

The enzyme-linked immunosorbent test (ELISA), PCR, and reverse-transcriptase PCR are the most used COVID-19 detection techniques. In compared to classical PCR, RT-PCR is the only method for detecting COVID-19, according to the World Health Organization and the American Center for Disease Control (ACDC) [43,44]. These detection technologies have several drawbacks, such as the requirement for trained staff and the usage of large volumes of expensive chemicals. Furthermore, traditional testing is time-consuming and unsuited for large-scale diagnosis. Despite the approaches’ great sensitivity, certain research publications have shown that they can provide misleading negative results.

PCR technique offers several advantages; however, there are also limitations when it comes to the pathogen identification. For example, the presence of a trace number of pathogens and their corresponding genetic content in collected specimens can lead to false negatives, whilst “inert” antibodies acquired from recovered patient’s samples might lead to false positives and be deceptive when making decisions. In addition, there are i) sample collection and transportation-related issues (such as sampling error, sample denaturation while transporting), ii) an insufficient number of clinical facilities and complicated device setup and maintenance issues (such as a lack of clinical facilities and qualified personnel to perform an extensive number of tests quickly during a pandemic), iii) resource shortage and inadequate quality of reagents (such as a lack of reagents and inadequate quality of reagents during a paned) [45,46].

With the advancement of nanobiotechnology, a new generation of devices, such as biosensors, are emerging that combine the criteria with novel needs in terms of noninvasive and simple specimen collection, transmission of test data after proper analysis, and immediate patient treatment or surveillance feedback. Electrochemical biosensors, for example, can be utilized as antigen fast detection devices. As a result, if they produce detection limits in the pico/nanomolar range, they are gaining a lot of interest in the COVID-19 management [47–49]. SARS-CoV-2 has been detected using a variety of electrochemical biosensors, including potentiometric, voltametric, impedimetric, and field-effect transistor (FET)-based devices. Because of biological binding events at their electrode surfaces, they assess changes in voltage, current, resistance, and conductance, respectively. The sensitivity and limit of detection (LOD) of a diagnostic technique are linked to the SARS-CoV-2 virus’s minimum viral load and infective virus dosage, which is a tough issue for a diagnostic platform [50,51].

**Figure 3:**
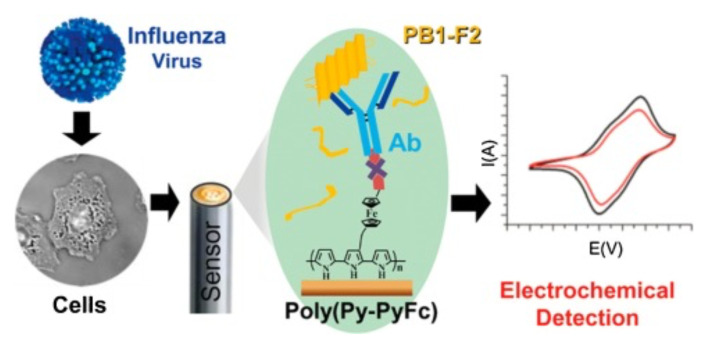
A graphical shown of the electrochemical detection of PB1-F2 protein of IAV (Figures are adapted with permission, Copyright 2014, American Chemical Society) [70].

There have been some recent investigations on the using area of biosensors to detect viruses. For example, field-effect transistor (FET) sensor (Figure 3) was created by Seo et al. (2020). In this study, 12 SARS-CoV-2 spike antibodies were used and they were attached to the graphene sheet that serves as the reporter part. This sensor was applied to detect the clinical samples of SARS-CoV-2 virus as well as the SARS-CoV-2 antigen. The system also displayed no detectable cross reactivity with Middle East Respiratory Syndrome coronavirus (MERS-CoV) antigen. As a result, their functionalized graphene-based sensor device enables the detection of the SARS-CoV-2 virus in clinic samples in a quick, and a very receptive attitude [52]. They established a graphene-based biosensing device (COVID-19 FET sensor) that SARS-CoV-2 spike antibody was functionalized for use as a SARS-CoV-2 viral detection platform in this inquiry. 1-pyrenebutyric acid N-hydroxysuccinimide ester (PBASE) which is an effective interface linkage agent employed as probe linker was used to immobilize SARS-CoV-2 antibody onto the designed device. Their COVID-19 FET sensor method can detect the target SARS-CoV-2 antigen with the LOD of 1 fg/mL. The antigen protein of SARS-CoV-2 was discovered in the transport medium used for nasopharyngeal swabs as well as produced SARS-CoV-2 virus, and also SARS-CoV-2 virus from clinical samples, indicating the possibility for clinical application. Furthermore, their sensor was able to differentiate the antigen of SARS-CoV-2 from the MERS-CoV. These findings suggest that a COVID-19 FET sensor based on an integration of SARS-CoV-2 spike antibody and graphene may detect SARS-CoV-2 virus in clinical samples with high sensitivity [52]. Some examples of SARS-CoV-2 detection strategies were summarized in Table 1.

Nowadays, localized surface-plasmon resonance (LSPR) has been a great option for detecting micro- and nanoscale analytes in instantaneously without the need of labels. Surface-plasmon resonance (SPR) was used to evaluate the physicochemical characteristics of SARS-CoV-2 spike protein in a recent study, and it was discovered that the SARS-CoV-2 spike glycoprotein has a far higher affinity for angiotensin-converting enzyme 2 (ACE2) than SARS-CoV spike protein. Pinals et al. used a single-walled carbon nanotube (SWCNT)-based optical sensor functionalized with ACE2 to detect the S-protein and SARS-CoV-2 virus-like particles [53]. With an 11 fM LOD, Raziq et al. created the first-time electrochemical sensor to detect SARS-CoV-2 nucleoprotein from nasopharyngeal samples of COVID patients; the benefit of this diagnosis approach is the rapidity of the detection procedure as well as the identification of important biomarkers [53,59]. Layqah et al. created an immunosensor for detecting MERS-CoV based on a competitive assay on an array of electrodes nanostructured with gold nanoparticles to allow multiplexed detection of various CoV in another investigation. The free virus in the sample and immobilized MERS-CoV protein with a fixed concentration of added antibody were in indirect competition at the biosensor’s base. At each phase, the ferro/ferricyanide redox couple’s reduction peak current was recorded. The test took 20 min to complete and had detection limits of 0.4 and 1.0 pg/mL for HCoV and MERS-CoV, respectively [60].

Biosensors are a promising technology since they are inexpensive, very sensitive, fast, and easy to use owing to their mobility. This study looks at newly reported electrochemical detection approaches that have enhanced responses to address issues with SARS-CoV-2 viral detection [61].

#### 1.2.2. Biodetection technologies for influenza virus

The influenza virus is a member of the *Orthomyxoviridae* viral family. This family includes the enveloped viruses with segmented negative-sense single-strand RNA segments in their genomes [62]. The influenza virus as an enveloped virus is particularly sensitive to the effects of the environment. It can, however, live for several hours in water at low temperatures (e.g., 20 °C) and considerably longer in water at high temperatures (e.g., 40 °C) depending on environmental factors (e.g., humidity and temperature) (up to several months). Lipid solvents and detergents are toxic to influenza viruses. Depending on the virus type, they are susceptible to a low pH and heat [63].

Influenza is a virus that causes illness. Electrical measurements and optical imaging might be used to distinguish a virus from structurally identical adenovirus and paramyxovirus. The suggested multiplexed platform offers significant promise for applicability to clinical applications because to its high sensitivity, selectivity, and ease of instrumentation [46]. A quick and reliable diagnostic test is required for early infection detection. The present approaches primarily focus on demonstrating the presence of certain virus antigens, stimulating an organism’s immunological response, proving virus enzymes, hemagglutination, and serologic tests based on the presence of specific antibodies [64]. Direct isolation of microorganisms is, of course, the most precise approach, but it is time- and expense-intensive. Early detection of an infection necessitates a reliable and, above all, quick diagnostic test with high sensitivity, ease of use, and cost sustainability.

##### 1.2.2.1 Biodetection technologies for influenza A viruses (IAVs)

Influenza A viruses (IAVs) are classified into three categories which are based on antigenic variations in the nucleoprotein (NP) and matrix (M) genes; type A, B, and C IAVs are the most common. The most clinically important influenza A viruses are categorized into subtypes which are based on antigenic variations in the neuraminidase (NA) and hemagglutinin (HA) genes [65, 66]. Numbers of studies have been focused on the detection systems for IAVs. For example, Dunajová et al. used screen printed carbon electrodes to build a highly selective impedimetric immunobiosensor with an ultrasensitivity and it was based on interaction with monoclonal antibodies for detecting IAVs [67]. They tested their system not only in the ideal buffered PBS solution where LOD was 0.79 fM and linear concentration range of virus was 0.18 f. to 0.18 nM but also in defibrinated horse blood. Limit of detection and the sensitivity of the sensor with and without human serum albumin (HSA) in buffered solution and horse blood were also determined. The lowest sensitivity was observed in the case of the sensor without HSA. If HSA was replaced by bovine serum albumin (BSA) different behavior was visible using electrochemical impedance spectroscopy. After the addition of higher virus nucleoproteins concentrations, the decrease of charge transfer resistance was detected in the case of BSA. LOD was the best in the case of the sensor without HSA in the buffered solution. In the horse blood sample, the LOD was almost 1000 times worse than in the previous case, however still good enough to be comparable with ELISA test [67].

In another study, Kumar et al. have shown a simple and quick technique for visual detection of several strains of IAVs (H1 to H16 subtypes) employing peptide nucleic acid (PNA) as a biosensor and pristine gold nanoparticles (AuNPs) as a reporter. This assay might aid national monitoring studies, particularly at the herd level, as well as the implementation of rapid control measures during early phases of an IAV pandemic. This visual assay’s simplicity and universality might be used to diagnose IAVs in humans [68].

Furthermore, Sayhi et al. developed a method for isolating and identifying the H9N2 subtype of the influenza A virus. They employed iron magnetic nanoparticles with an antimatrix protein 2 antibody to isolate the influenza virus from serum fluid. Then, using the fetuin-hemagglutinin interaction, gold nanoparticles were linked to an electrochemically detectable label to detect the virus [69].

During the viral cycle, Miodek et al. established an immunosensing technique based on the PB1-F2 forms of IAV protein (Figure 3). Antibodies specific for PB1-F2 forms were bound onto the polymeric matrix formed onto the Au electrode for this purpose. The researchers employed cyclic voltammetry (CV) to detect monomeric or oligomeric PB1-F2 forms of IAV protein in that study [70].

Another electrochemical genosensor was created to determine oligonucleotide sequences associated to H5N1 strain of avian influenza virus. The Au electrode was modified using redox active monolayers such as (dipyrromethene)_2_Cu(II) and (dipyrromethene)_2_Co. Then, using ethyl(dimethylaminopropyl)carbodiimide (EDC) / N-hydroxysuccinimide (NHS) linkers, a 20-mer probe (any probe terminates with an NH_2_-NC_3_ functional group and any probe is 25 nucleotides long) was connected covalently to the electrode surface from the redox-active monolayer’s carboxylic groups. The electron transport was prevented during hybridization reactions between the NH_2_-NC_3_ probe and the target sets. The redox active layer was covered. As a result, there was a reduction in the concentration of the analyte (target DNA) [71]. In another study, Kamikawa et al. presented another study in which they created a direct-charge biosensor based on electroactive magnetic (EAM) nanoparticles (Fe_2_O_3_) for the identification of the IAV H5N1 surface glycoprotein Hemagglutinin (HA). Polyaniline-coated was immunofunctionalized using antibodies against the target HA in that investigation. The anti-HA–EAM complexes successfully separated HA which is from mouse serum matrix by acting as an immunomagnetic separator. H5 and H1 glycoproteins were detected using a biosensor that was made. Consequently, the created biosensor’s sensitivity for detecting recombinant H5 was reported to be 1.4 M in 10% mouse serum, and it had a high selectivity for H5 when compared to H1 [72]. Furthermore, Veerapandian et al. developed an electrochemically based dual-sensor device made up of methylene blue electro-adsorbed graphene oxide nanostructures customized with monoclonal antibodies against H5N1 and H1N1 HA proteins. The developed dual immunosensor was simple to build bio-based analysis system with quick analysis time (less than 1 min), and highest repeatability. In addition, it provided excellent sensitivity (picomolar level) for H5N1 and H1N1 HA proteins. A simple and sensitive influenza test is particularly advantageous in the light of the requirement for quick diagnosis of influenza risks in poultry operations and related severe consequences [73].

Microelectrode array covered with coiled-coil peptide (CCP) was used to build an electrochemical biosensor to analyze antibodies, according to Arya et al. In conclusion, that electrode with a self-assembled monolayer of CCP containing the HA-antibody specific peptide sequence can be used as an ultrasensitive electrochemical immunosensor for detecting HA-antibodies [74]. Some examples of IAVs detection strategies were summarized in Table 2.

The seasonal influenza A virus subtypes (H1N1 and H3N2) cause 3–5 million human severe illnesses and between 290,000 and 650,000 fatal cases each year, predominantly in the elderly and immunocompromised, as well as small children [75, 76]. To effectively manage future epidemics, it was critical to enhance H1N1 and H3N2 virus diagnostics. According to the information provided, biosensors are an ideal diagnostic method with simple sample collection and preparation, highlighting the technique’s strong potential as a highly effective point-of-care diagnostic platform for the rapid, accurate, and specific detection of various lAVs viral pathogens.

#### 1.2.3 Biodetection technologies for hepatitis virus

Hepatitis is an inflammation-causing illness of the liver. It can be self-limiting or progress to fibrosis (scarring), cirrhosis, or cancer of the liver. Hepatitis is caused mostly by hepatitis viruses, although it can also be caused by infections, toxic substances (such as alcohol and some medications), and autoimmune diseases. There are five kinds of hepatitis viruses: A, B, C, D, and E. These five categories are the most alarming because of the amount of disease and death they cause, as well as the potential for outbreaks and epidemic transmission. Types B and C, particularly, are the primary causes of liver cirrhosis and cancer, affecting hundreds of millions of people (World Health Organization, hepatitis, accessed 2020) [77].

##### 1.2.3.1 Biodetection technologies for hepatitis B virus

Hepatitis B virus (HBV) is one of the common illnesses, with cirrhosis and malignant liver development causing over a million deaths each year. Furthermore, 15% percent to 40% of infected individuals may develop liver failure, cirrhosis or hepatocellular carcinoma and 15% percent to 25% of infected patients will die [78]. There is a serological profile over the 2 billion people indicating previous or present HBV infection, and 360 million people with the chronic HBV-related liver disease, according to the estimates. Patients with a serum viral load of >10^5^ copies/mL are at an extremely high risk (risk evaluation of viral load increase and associated liver disease/cancer-hepatitis B virus). As a result, detecting and monitoring HBV in the early stages of infection is critical [11,79]. Immunoassay and PCR are now the most widely used clinical diagnostic techniques for HBV detection. Immunoassays are based on serological procedures that target viral antigens or antibodies and can reach 100 percent accuracy and selectivity. The immunoassay approach, on the other hand, does not yield quantitative data, and detection is restricted by serological response [80]. Furthermore, because PCR needs good heat cycle management and hence greater apparatus expenses, it comprises of 20–40 repeated cycles to reach a measurable DNA concentration. The hybridization process in PCR is also known to create mistakes. As a result, in comparison to standard clinical diagnostic procedures, it is important to build a detection approach that has the following characteristics: cost-effectiveness, rapid response, portability, and high sensitivity [81]. This chapter looks at the current state of biosensor research with respect to efficient, specific, and rapid detection of HBV. For example, Zhang et al. created an electrochemical DNA biosensor to detect HBV DNA fragments. The biosensors are based on the covalent immobilization of HBV genes-related 21-mer single-stranded DNA (ssDNA) on a modified glassy carbon electrode (GCE) [82]. Using potentiodynamic polarization voltammetry and (CdL2)^2+^ that it is a novel mediator, the hybridization between both the probe and its matching ssDNA was examined. To test the selectivity of the constructed electrochemical DNA biosensor, experiments were conducted with noncomplementary oligonucleotides [70]. In another study, Li et al. utilized gold@platinum nanorods (Au@Pt) as the signal probes in lateral flow biosensors (LFBs) to detect hepatitis B viral DNA (HBV-DNA). In absence of hydrogen peroxide, the oxidation of 3,3′,5,5′-tetramethylenebenzidine (TMB) to a blue substrate may be effectively catalyzed by Au@Pt nanorods with improved oxidase-like activity [83]. Moreover, Hashimoto et al. described a photolithography-based microfabricated disposable-type electro-chemical nucleic acid sensor to detect HBV genomic DNA. The nucleic acid sensor had a strong correlation with competitive PCR when HBV-DNA isolated from patients’ serum was measured in the range of 10^4^–10^6^ copies/mL. The findings revealed that the nucleic acid sensor can accurately and quantitatively detect HBV DNA in serum [84].

In order to construct peptide nucleic acid-quartz crystal microbalance (PNA-QCM) biosensors to monitor of HBV hybridization experiment, Yao et al. employed PNA probes instead of DNA probes. The PNA probes are more effective and selective at combining target sequences than DNA probes. PNA probe was created and immobilized on biosensor’s surface to replace the traditional DNA probe to detect the HBV genomic DNA without the need for PCR amplification [85]. Lee et al. showed the use of low wave mode Surface acoustic wave (SAW) immunosensors under aqueous settings to detect HBs antibodies. To identify selective binding of HBs antibody to conjugated HBsAg, the resonance frequency shift was observed. Without any pretreatment, SAW immunosensor can analyze the HBs antibodies in the whole blood samples [86]. Using an HBsAg and a secondary antibody, Chung et al. exploited surface plasmon resonance (SPR) to analyze captured human HBV antibodies. The SPR biosensor’s detection limit for medical diagnosis was comparable to that of a commercial ELISA kit [87]. Walters et al. established a hybrid biosensor based on graphene resistor modified with self-assembled graphene-AuNPs (HBsAg) was used to detect hepatitis B surface antigen. The graphene sensor was effectively used to detect HBsAg, a biomarker for HBV that could be acute or chronic. Any biomarker of interest might be detected to use the hybrid biosensor platform [88]. Some examples of HBV detection strategies were summarized in Table 3.

##### 1.2.3.2 Biodetection technologies for hepatitis C virus

Hepatitis C virus (HCV) infection causes liver disease such cirrhosis and hepatocellular carcinoma. HCV is found in around 3% of the world’s population. As a result, HCV infection is now considered a public health risk [90]. Various studies have focused on the development of new detection systems based on biological molecules. For example, Wu et al. created a highly selective and sensitive fluorescence biosensor for HCV which detects the virus using -FeOOH and exonuclease III-assisted signal amplification. Transmission electron microscopy (TEM) was used to image-FeOOH nanosheets. For the first time, a low surface fluorescence sensing platform based on enzyme-aided signal enhancement and magnetic separation was constructed to boost the sensitivity of HCV DNA [91]. Furthermore, Srisomwat et al. have suggested a sensitive point-of-care sensing platform for HIV/HCV cDNA detection, and this uses the alternating-current electroluminescence (ACEL) approach. The DNA detection was made possible in a label-free manner by a conductance-based light emission that was regulated by the hybridization of a pyrrolidinyl PNA probe with the DNA target. The presence of the target DNA resulted in higher proton conductivity, which enhanced electroluminescence. The findings of testing the positive and negative samples from the patient’s serum agreed with the results of the commercial kit based on the real-time PCR technique, demonstrating the created sensor’s high sensitivity and specificity. Their point-of-care sensing technique offered rapid, simple to operate, affordable, portable, and the ability for real-time monitoring, for the sensing application [92].

#### 1.2.4 Biodetection technologies for human immunodeficiency virus (HIV)

Human immunodeficiency virus (HIV) is one of the most popular dangerous retroviruses. HIV infection produces acquired immunodeficiency syndrome, which is called as AIDS, that it is one of the deadliest illnesses in the world. AIDS, as one of the century’s major issues, is quickly spreading. According to the data, 36.9 million individuals worldwide were surviving with HIV. At the end of 2017, with the global HIV generality of 0.8 percent among adults [93]. Such as these conditions, impedimetric analysis is beneficial for identification of HIV related genes (2007) since the viruses infect hundreds to thousands of individuals globally and these infected persons create antibodies in blood against to the viruses [94]. Different HIV testing kits have been developed to detect antibodies directed toward different components of HIV as AIDS has become a global public health problem. These tests have a significant drawback in that they are unable to identify HIV antibodies during the early stages of viral infection. As a result, a variety of biosensors for early HIV infection diagnosis have been developed to meet this problem. The use of nanomaterials to improve the sensitivity and accuracy of sensing systems has been the subject of many these investigations [93].

A lot of studies have been developed to identify infection using DNA hybridization or an assay for HIV-related proteins; however, there are fewer reports on the detection of viral particles in the literature. In reality, numerous indirect approaches for HIV early detection have been established. The detection of HIV type 1 or HIV type 2 antibodies, viral DNA (RNA), viral p24, p17, HIV-related enzymes, and CD4^+^ T cells counts are some of the procedures used. Nanotechnology has opened new opportunities in the development of these biosensing tests. The increased sensitivity of biosensors is due to the larger surface-to-volume ratio, electrical, and optical characteristics of nanostructures. Carbon nanostructures, quantum dots, nanoclusters, metallic and metal oxide nanoparticles are just a few of the well-known materials that are being considered as potential possibilities for developing extremely sensitive HIV biosensors [93].

Affinity biosensing technique, magnetic nanoparticles were used as a label for diagnostic purposes. These particles are used to improve biomolecule or particular cell separation, concentration, and identification. There are two steps for biomagnetic separation. The first phase is labeling and tagging the item of interest that it needs to be separated from each other with a magnetic material, followed by the separation of these tagged species using precise electromagnetic separation devices. The aim of analysis is that magnetic nanoparticles are used as a linker or binder between the analyte and the label. Because the antibodies are particular in their function, they only bind to the antigen that matches it, resulting in extremely exact and accurate results [95].

## 2. Conclusion

Treatment is becoming a highly essential problem for prevention, which also depends on early and correct diagnosis, with the rising number of patients with infectious illnesses and the accompanying mortality [96]. With the global pandemic of COVID-19, the importance of rapid diagnosis kits has emerged once again. Biosensor technologies have the ability to meet these criteria owing to an interdisciplinary mix of methodologies from chemistry, nanotechnology and medical research [11]. New identification components have been investigated to enhance recognition in biosensing because of the need for quick diagnosis and advancements in more stable, selective, and cost-effective biosensor technology [68]. The demand for research into the development of diverse sensor platforms based on electrochemistry and optics is growing by the day. Furthermore, there is a growing interest in research into developing novel materials, altering surface chemistry, and enhancing biological molecule immobilization techniques. These point-of-care gadgets, once widely available, will decentralize the disease’s diagnosis and monitoring to the community level. As a result, individuals will be better prepared to battle a worldwide epidemic, and the healthcare industry’s burden will be greatly reduced. Because of its portability, speed, and durability, it will significantly enhance access to cutting-edge technology in industrialized countries, creating a luxury. Similarly, in resource-constrained nations, the low cost and ease of use will dramatically enhance access to previously unattainable technologies. Interest in studies on the creation of various sensor platforms based on electrochemistry and optics is increasing day by day. In addition, the interest in studies on synthesizing new materials, changing surface chemistry and improving the immobilization strategies of biological molecules is increasing nowadays. As a result, nanobiotechnology and sensing methods will be used in the future to tackle problems on both sides of the coin [97].

## Figures and Tables

**Table 1:** Biodetection technologies for COVID-19 (SARS-CoV-2) and its proteins.

Target analyte	Sensor type	Reporter type	Detection mode	Detection limit	Sample	Ref.
SARS-CoV-2 SP	Electrochemical	Antibody on graphene sheets	FET	1 fg/mL in PBS and 100 fg/mL	Clinical transport medium	[52]
SARS-CoV-2	Electrochemical	Antibody on graphene sheets	FET	LOD: 1.6 × 10^1^ pfu/mL in culture medium and LOD: 2.42 × 10^2^ copies/mL	Clinical samples	[52]
SARS-CoV-2 SP	Optical	SWCNTs noncovalently functionalized with ACE2	Fluorescence	Approx. 10^4^–10^6^ (viral particles) / approx. 10^−8^ M (S RBD)	Saliva and viral transport medium	[53]
SARS-CoV-2	Electrochemical	Magnetic beads as support of immunological chain	Voltammetry	19 ng/mL for spike protein and 8 ng/mL for nucleocapsid	Untreated saliva	[54]
SARS-CoV-2 SP	Electrochemical	BSA/AB/f-GO/GCE	Voltammetry	1 ag/mL	Saliva	[55]
SARS-CoV-2 SP	Electrochemical	BSA/AB/f-GO/SPE	Voltammetry	1 ag/mL	Saliva	[55]
SARS-CoV-2-RBD	Electrochemical	MIP on a microporous gold screen-printed electrode	EIS	0.7 pg/mL (20 fM)	Saliva	[56]
Recombinant IgG antibody to SARS-CoV-2 spike glycoprotein S1	Electrochemical	SARS-CoV-2-RBD on ZnO NWs modified paper-based electrodes	EIS	Tested for 10 ng/mL, 100 ng/mL, 1 μg/mL	Human serum samples	[57]
SARS-CoV-2 proteins	Electrochemical	GO/Gr	FET	~8 fg/mL	Throat swab buffer solution	[58]

SP: spike protein; PBS: phosphate-buffered saline; FET: field-effect transistor; SWCNTs: single-walled carbon nanotubes; BSA/AB/f-GO/GCE: bovine serum albumin, SARS-CoV-2 spike antibody and a functionalized graphene oxide modified glassy carbon electrode; BSA/AB/f-GO/SPE: bovine serum albumin, SARS-CoV-2 spike antibody and a functionalized graphene oxide modified screen-printed electrode; SARS-CoV-2-RBD: receptor-binding domain of severe acute respiratory syndrome coronavirus 2; MIP on MP-Au-SPE: molecular imprinted polymer on a macroporous gold screen-printed electrode; EIS: electrochemical impedance spectroscopy; ZnO NWs: zinc oxide nanowires; GO/Gr: graphene oxide-graphene.

**Table 2:** IAVs detection using bio-based technologies.

Target molecule	Sensor type	Reporter type	Detection mode	Detection limit	Sample	Ref.
IAVs	Electrochemical	Antibody on SPE	EIS	0.79 fM	Buffered solution and horse blood	[67]
Multiple strains of IAVs	Optical	PNA and AuNPs	Colorimetric	2.3 ng for IAV RNA	Avian clinical samples	[68]
PB1-F2	Electrochemical	Antibody on Conductive PPymodified with ferrocenyl groups	CV	0.42 nM for monomeric PB1-F2 and 16 nM for oligomers	Lysates of infected cells	[70]
H5N1	Electrochemical	20 mer probe on (dipyrromethene)2Cu(II) and (dipyrromethene)2Co(II)	OSWV	1.39 pM	-	[71]
HA-H5N1	Electrochemical	Antibody on EAM	CV	9.4 pM	Mouse serum	[72]
HA for H5N1 and H1N1	Electrochemical	Antibody on GO	DPV	9.4 pM for H1N1 and 8.3 pM for H5N1	-	[73]

EIS: electrochemical impedance spectroscopy; SPE: screen printed electrode; PNA: peptide nucleic acid; AuNPs: gold nanoparticles; PPy: polypyrrole; CV: cyclic voltammetry; OSWV: Osteryoung square wave voltammetry; HA: hemagglutinin from the influenza A virus H5N1 (A/Vietnam/1203/04); EAM: electrically active magnetic nanoparticles; GO: graphene oxide nanostructures; DPV: differential pulse voltammetry.

**Table 3:** Hepatitis B Virus detection using bio-based detection technologies.

Target molecule	Sensor type	Reporter type	Detection mode	Detection limit	Sample	Ref.
HBV DNA Fragments	Electrochemical	21-mer single-stranded DNA and CdL_2_	DPV	7.19 × 10^−9^ mol/L	-	[82]
HBV DNA	LFBs	Au@Pt	Colorimetric	8.5 pM	-	[83]
HBV DNA	Piezoelectric	PNA	QCM	8.6 pg/L	Human blood samples	[85]
HBs antibody	SAW	Hepatitis B surface antigen	Love wave	10 pg/μL	Whole blood samples	[86]
hHBV	SPR	hHBV antigen	SPR	9.20 nM for direct assy 4.39 nM for sandwich assay 0.64 nM for PAP method	Serum	[87]
HBsAg	Electrochemical	Graphene-AuNPs	Resistance	50 pg/mL	-	[88]
HBV DNA	Electrochemical	AuNCs	EIS	0.1 fM	Blood serum	[89]

CdL_2_: diaquabis[N-(2-pyridinylmethyl) benzamide-κ2 N,O]-cadmium(II) dinitrate {[CdL2(H2O)2](NO3)2, where L = N-(2-pyridinylmethyl) benzamide}; DPV: differential pulse voltammetry; LFBs: lateral flow biosensors; Au@Pt: gold@platinum nanorods; QCM: quartz crystal microbalance; PNA: peptide nucleic acid; SAW: surface acoustic wave; SPR: surface plasmon resonance; hHBV: human hepatitis B virus; AuNPs: gold nanoparticles; HBsAg: hepatitis B surface antigen; AuNCs: gold nanocrystals.
